# Mobitz Type-2 Heart Block After a Bee-Sting

**DOI:** 10.7759/cureus.11856

**Published:** 2020-12-02

**Authors:** Abdul Chaudry

**Affiliations:** 1 Cardiology, University of North Carolina, Chapel Hill, USA

**Keywords:** bee sting, heart block, kounis syndrome

## Abstract

We report the case of a patient who developed symptomatic bradycardia and Mobitz type 2 heart block one week after a single bee sting. This required implantation of a permanent pacemaker. The patient had no significant past medical history, and previous electrocardiogram (ECGs) did not show heart block or bradycardia. He has been physically active in the past and denied any such symptoms. We presume bee sting to be the cause of his symptomatic bradycardia and heart block. We also think that his heart block was reversible and has since resolved, as his most recent device check showed minimal V-pacing. The cause of his heart block can be either Kounis syndrome or Apamin-mediated calcium channel block. A detailed discussion is done separately.

## Introduction

Bee-stings can present in a variety of ways, ranging from simple localized pain and inflammation to fatal cardiovascular complications. Some potential cardiovascular complications include atrial fibrillation, ventricular fibrillation, Takotsubo cardiomyopathy, myocardial infarction, and heart block [1-5]. Mobitz type-2 heart block is a type-2 AV nodal block that can prove fatal if untreated. Here we report a patient who developed presyncope and Mobitz type-2 heart block after getting stung by a bee.

## Case presentation

**Figure 1:**
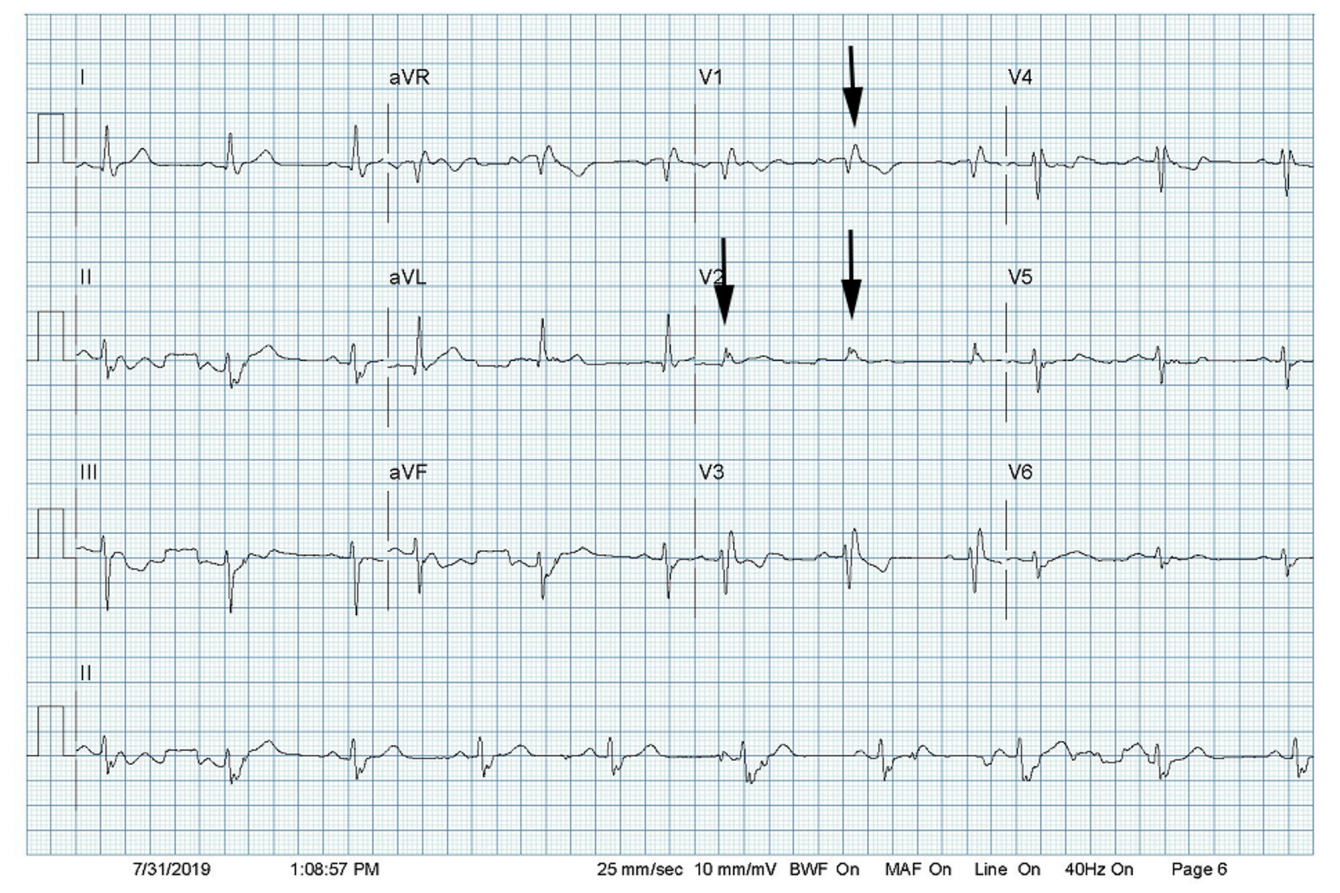
Resting electrocardiogram (ECG) after admission Arrows show right bundle branch block.

A 67-year-old male living in eastern Pennsylvania presented to the emergency room with dizziness. He denied any significant past medical history, but reported a bee sting one day prior to arrival. His vitals and physical examination were normal except for a heart rate of 42 bpm. An electrocardiogram (EKG) was done, which showed the right bundle branch block (Figure 1). Serum troponin was negative. The carotid duplex was normal with patent carotid arteries bilaterally. Lyme serology was obtained, which returned negative. His echo revealed a left ventricular ejection fraction of 60% with grade I diastolic dysfunction. He reported taking a dose of Diphenhydramine for bee sting and denied any other medications.

**Figure 2:**
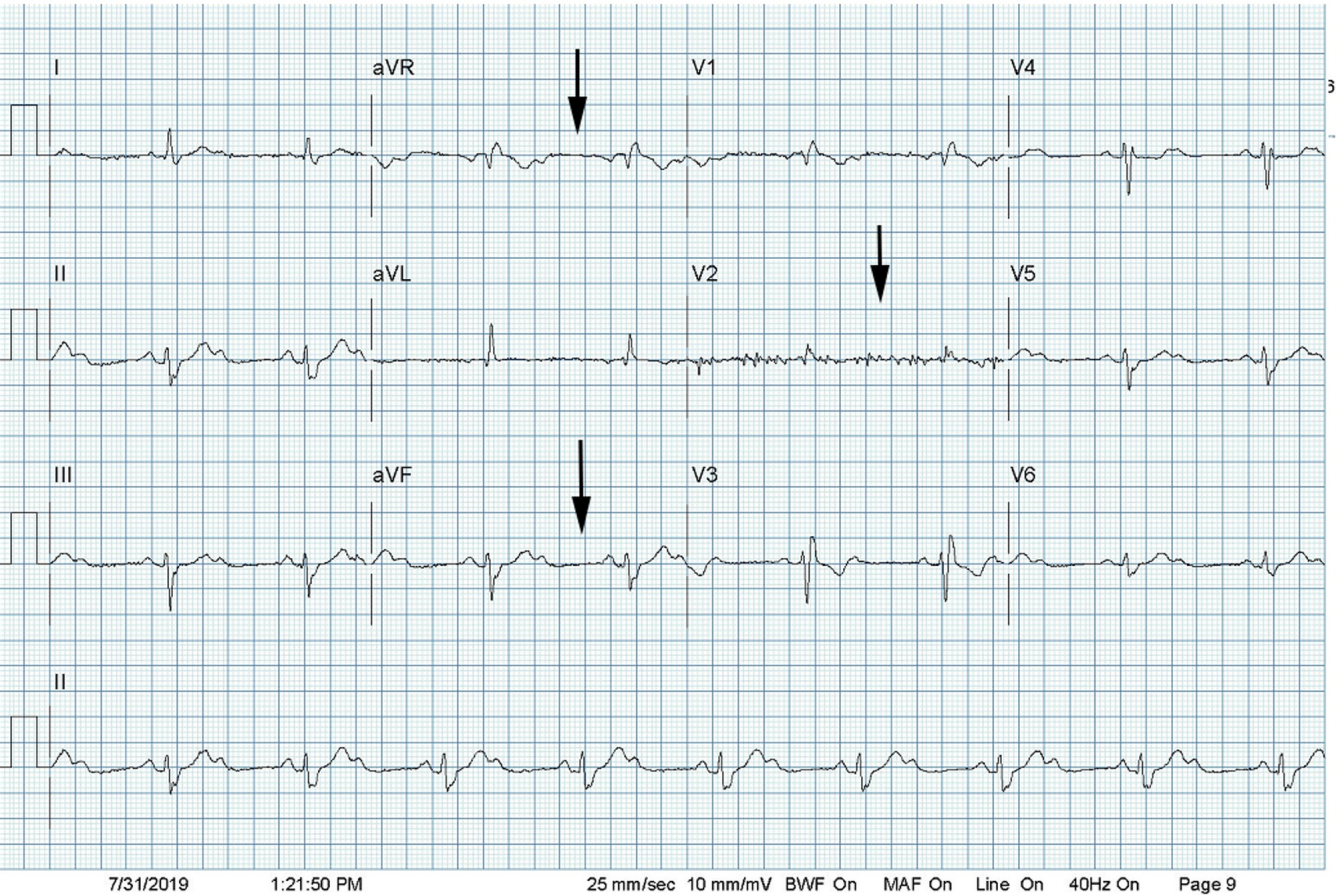
Mobitz type-2 heart block

Shortly thereafter, the patient developed shortness of breath and dizziness. A treadmill nuclear stress test was ordered, which was discontinued after 4 minutes and 15 seconds because the patient developed a 2:1 Atrioventricular block (Mobitz type 2) (Figure 2). His ventricular rate was noted at 55 bpm at that time.

**Figure 3:**
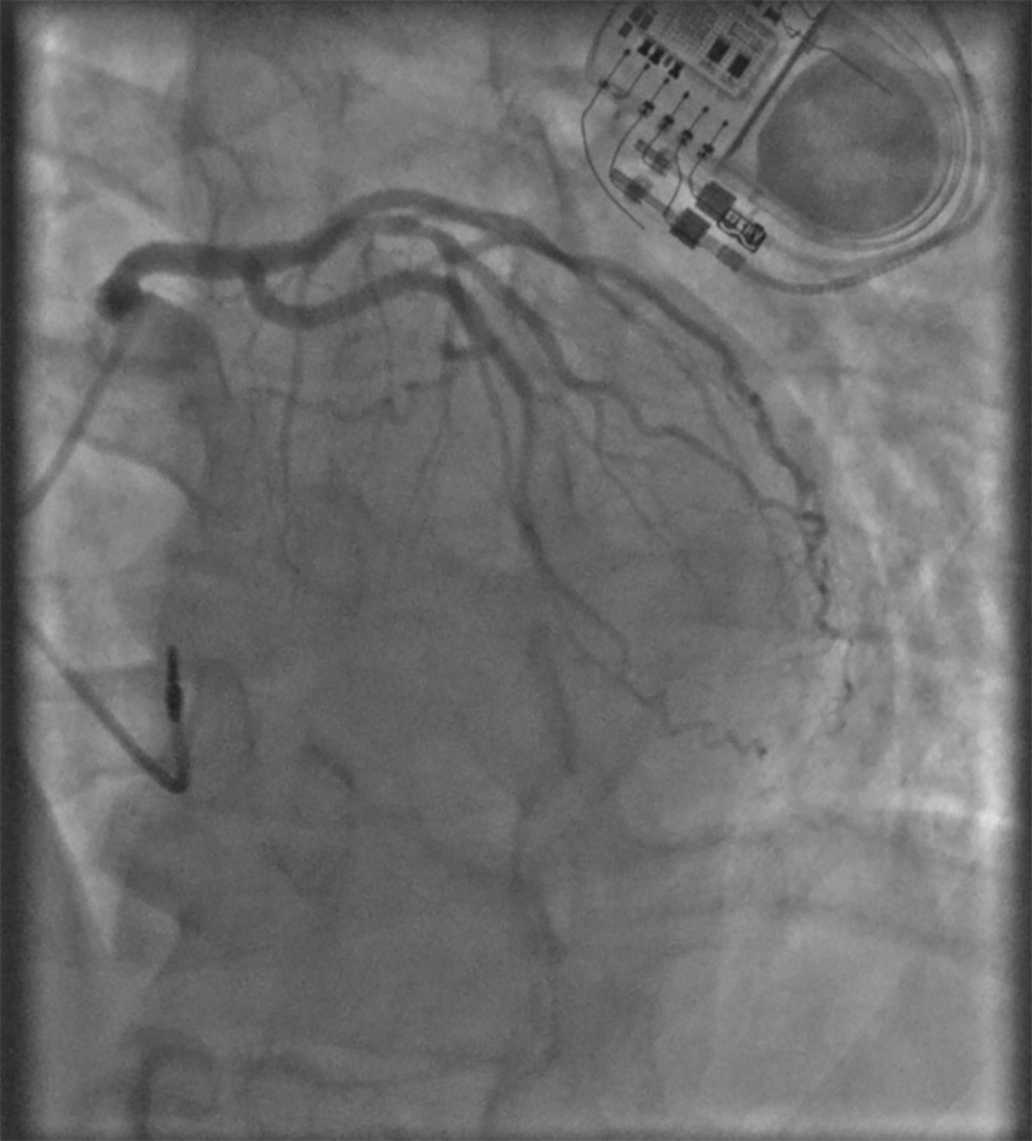
Coronary catheterization; 40% stenosis in proximal to mid-left anterior descending artery

**Figure 4:**
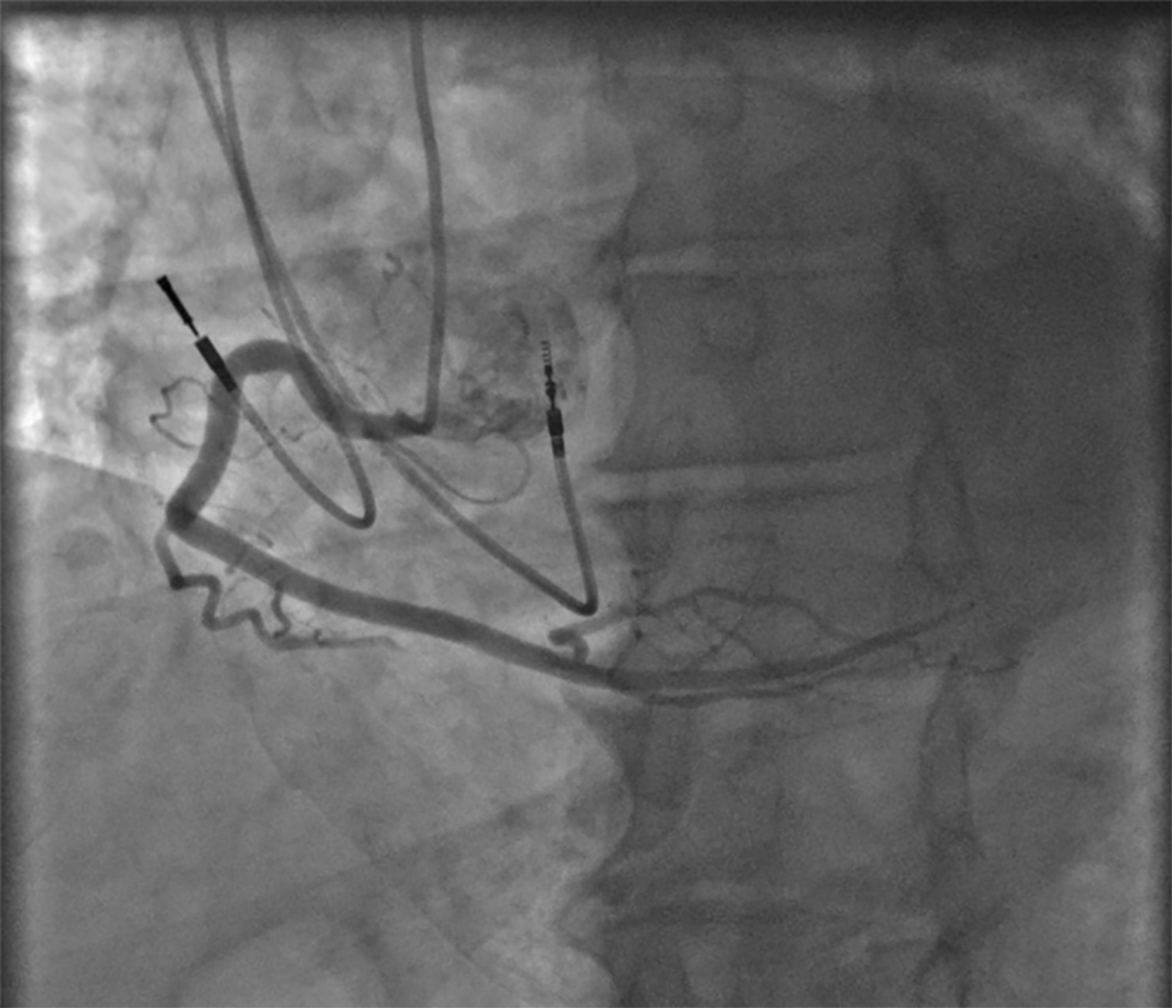
Coronary catheterization; 20% stenosis in proximal right coronary artery (RCA)

Subsequently, the patient was implanted with a dual-chamber (DDD) pacemaker, with complete resolution of symptoms. Two weeks after pacemaker implantation, his device check showed 50% A-paced and 8% V-paced. Cardiac catheterization three weeks after discharge showed 40% tubular stenosis in proximal to the mid-left anterior descending artery with mildly elevated left ventricular end-diastolic pressure, and 20% stenosis in the proximal right coronary artery (Figures 3, 4). The patient returned for device check three months after implantation, which showed 86% A-paced and 1% V-paced, and no recurrence of symptoms.

## Discussion

This is a case of possible Kounis syndrome after a single bee sting. The patient was previously asymptomatic and ended up getting a permanent pacemaker after two episodes of syncope and Mobitz type 2 heart block. Although there are a few case reports of heart block secondary to bee sting reported in literature, this is a rare case reported in the United States (Table 1).

**Table 1:** Previously reported cases of heart block with bee-sting

Case reported	Geographical region	Age of patient/gender	Presentation	Outcome
Vishwanath et al. [6]	Nepal	25/ Male	Myocarditis and Mobitz type-1 heart block	Discharged after 23 days in the hospital.
Gupta et al. [5]	India	55/ Male	Presyncope and Mobitz type-2 heart block	The patient was discharged on a temporary pacemaker later replaced with a permanent pacemaker.

Composition of bee venom

Bee venom is a complex mixture of proteins, amino acids, phospholipids, sugars, and biogenic amines. Melittin is the primary and most toxic compound in bee venom, constituting 50-60% of the whole venom [7]. It is responsible for catecholamines release. Metalloproteinases mediate hypersensitivity reactions and have several serious cardiac effects, including coronary vasospasm and plaque destabilization [8]. MCD and apamin are also present in bee venom in large amounts associated with bee venom's cardiac effects.

Possible causes of heart block

1. Kounis Syndrome Type 2

The clinical presentation and sequence of events all predict Kounis syndrome. Kounis syndrome was first reported in the American Heart Journal in 1950 with an allergy to penicillin. It has also been called "allergic” myocardial infarction, in which histaminergic mast cell degranulation leads to vasospasm [9]. In this case report, we present a similar case of possible Kounis syndrome with vasospasm of the right coronary artery leading to reversible Mobitz 2 heart block which is caused by an allergic reaction from the contents of bee venom. As previously described in the literature, Kounis syndrome is reversible, and coronary catheterization usually fails to detect any significant blockage. However, the complications arising from Kounis syndrome are permanent [5, 10].

2. Apamin-Mediated Block of Calcium-Activated Potassium Channel

A study done by Hussein et al. showed the effects of bee venom on cardiac muscle in toads. Application of the venom with either of three doses used (0.5, 1, and 2 ug/ml) resulted in severe bradycardia and an increase in the P-R interval, and increasing the R-wave amplitude [11, 12]. Apamin is a bee venom polypeptide and has been associated with the blockade of calcium-dependent potassium channels. It tightly binds to the slow calcium channels, blocking the heart muscle's slow action potential [12]. However, more recent studies show that apamin do not inhibit calcium, sodium, and potassium currents in the human heart [13]. Thus, the role of Apamin in the effects of bee stings remains controversial.

## Conclusions

Bee stings can cause cardiac complications in patients through either Kounis syndrome or blockade of calcium-activated potassium channels. Heart block can be a serious and fatal consequence of bee stings, and physicians should be aware of severe outcomes of such an event. It is essential to rule out vasospasm by doing appropriate cardiac studies immediately and counsel patients to watch for any cardiac symptoms for up to a few weeks when a patient presents with such history.
